# Secondary sphere interactions modulate peroxynitrite scavenging by the E2 domain of amyloid precursor protein†

**DOI:** 10.1039/d4dt02552k

**Published:** 2024-12-05

**Authors:** Eli C. Zuercher, Andrew T. Poore, Devendra Prajapat, Joseph Palazzo, Alana Thomas, Caitlin Birthright, Jack Lawrence, Ming Chen, Shiliang Tian

**Affiliations:** a Department of Chemistry, Purdue University West Lafayette Indiana 47906 USA sltian@purdue.edu

## Abstract

Peroxynitrite (ONOO^−^) is a highly reactive nitrogen species that can cause significant damage to proteins, lipids, and DNA. Various enzymes, including metalloenzymes, play crucial roles in reducing ONOO^−^ concentrations to protect cellular components. While the interaction of ONOO^−^ with heme proteins is well known, the reduction by Cu-containing proteins is less studied. Amyloid precursor protein (APP), implicated in Alzheimer's disease, has an E2 domain that binds copper ions with a dissociation constant of *K*_D_ ∼ 10^−12^ M and is proposed to be involved in iron homeostasis, copper trafficking, and oxidative stress response. Our recent studies using EXAFS, UV-Vis, and EPR spectroscopy revealed a previously unidentified labile water ligand in the Cu(ii) site of the E2 domain, suggesting reactivity with anionic substrates like ONOO^−^. Experimental data showed that Cu(i)-E2 reduces ONOO^−^ at a significant rate (1.1 × 10^5^ M^−1^ s^−1^), comparable to native peroxynitrite scavengers, while maintaining active site integrity through multiple redox cycles. This study further investigates the mechanism of ONOO^−^ reduction by Cu(i)-E2 using the Griess assay, demonstrating that reduction occurs *via* single electron transfer, forming nitrite and nitrate. This process aligns with previous findings that Cu(i)-E2 is oxidized to Cu(ii)-E2 upon ONOO^−^ reduction. Mutations at Lys435, affecting secondary sphere interactions, revealed that factors beyond electrostatics are involved in substrate recruitment. MD simulations suggest that steric hindrance from a newly formed hydrogen bond also plays a role. Understanding ONOO^−^ reduction by the E2 domain of APP expands our knowledge of copper proteins in mitigating oxidative stress and elucidates their physiological and pathological roles, particularly in Alzheimer's disease.

## Abstract 2

Peroxynitrite (ONOO^−^) is a reactive nitrogen species causing cellular damage. The copper-binding E2 domain of amyloid precursor protein reduces ONOO^−^*via* single-electron transfer, highlighting its role in oxidative stress and Alzheimer's disease.

## Graphical abstract

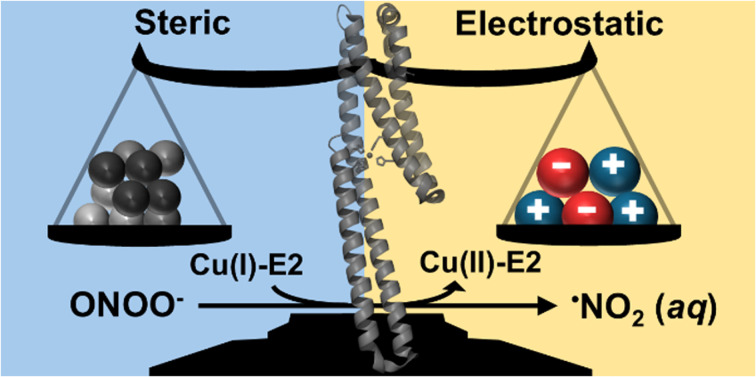

## Introduction

**Scheme 1:**
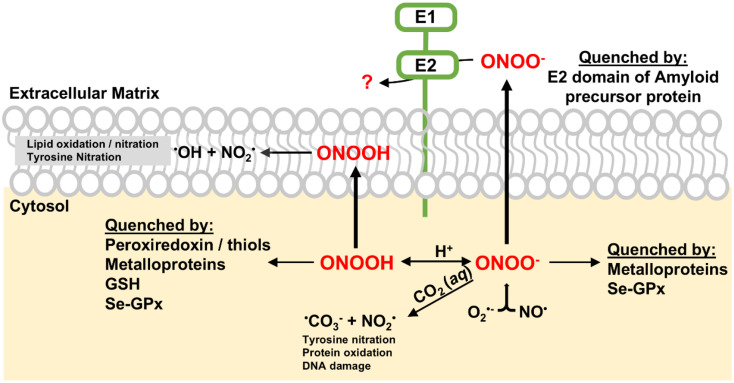
Possible pathways and targets of ONOO^−^. ONOO^−^ reacts rapidly with CO_2_, leading to the formation of NO_2_ and CO_3_˙^−^ radicals. NO_2_ can react with biomolecules, resulting in nitrated compounds. Alternatively, ONOOH can undergo slow homolysis to generate ˙OH and NO_2_ radicals, causing lipid oxidation and tyrosine nitration. ONOO^−^ can be quenched by various proteins such as peroxiredoxin (Prx), heme-containing proteins and selenium-containing protein glutathione peroxidase (Se-GPx), mitigating its cellular damage.

Peroxynitrite (ONOO^−^) is a reactive nitrogen species (RNS) generated by the rapid reaction between nitric oxide (NO) and superoxide (O_2_^−^) at diffusion-controlled rates (∼10^10^ M^−1^ s^−1^).^1^ Both NO and O_2_^−^ are free radicals produced in biological systems. ONOO^−^ is a potent oxidant and nitrating agent capable of causing significant damage to cellular components, including proteins, lipids and DNA (Scheme 1).^2^ This makes it a pathogenic mediator in various diseases such as various cardiovascular diseases, neurological diseases, and cancer.^2–4^

A notable feature of peroxynitrite is its ability to diffuse readily across lipid membranes. The calculated permeability coefficient for ONOO^−^ is 8.0 × 10^−4^ cm s^−1^, which is comparable to that of H_2_O and is approximately 400 times greater than that of superoxide.^5^ This high permeability allows ONOO^−^ to diffuse over distances greater than the diameter of a typical cell, making it an extremely effective oxidant capable of causing damage even far from its origin. To mitigate the cellular damage caused by ONOO^−^, several enzymes, including metalloenzymes, play key roles in reducing peroxynitrite *in vivo*.^4^ Peroxiredoxins (Prxs) are a family of thiol-specific antioxidant proteins that reduce ONOO^−^ to nitrite.^6,7^ They utilize cysteine residues in their active sites to catalyze this reduction, forming disulfide bonds that are subsequently reduced by thioredoxin.^8^ The selenium-containing protein glutathione peroxidase (Se-GPx) also quickly reduces ONOO^−^ to nitrite catalytically at the expense of glutathione (Scheme 1).^9^ Key metalloproteins involved in ONOO^−^ reduction are heme-containing proteins such as hemoglobin, myoglobin, cytochrome c, cytochrome c oxidase, and catalase.^10–14^ Hemoglobin and myoglobin can catalyze the isomerization of ONOO^−^ and convert it to nitrate by recombination of caged Fe(iv)=O and nitrogen dioxide radical (NO_2_) intermediates.^10,11^ Cytochrome c, an essential component of the electron transport chain, reduces ONOO^−^ to NO_2_, leading to the nitration of tyrosine residues.^12^ Mitochondrial cytochrome c oxidase has been reported to catalyze the conversion of ONOO^−^ to NO.^13^ Catalase, primarily known for decomposing hydrogen peroxide, can catalytically scavenge ONOO^−^.^14^ These heme-containing proteins, through their unique mechanisms, help maintain cellular redox balance and protect cells from the damaging effects of ONOO^−^, highlighting their importance in cellular defense against oxidative and nitrosative stress.

Compared to the well-known reactivity between ONOO^−^ and heme proteins, ONOO^−^ reduction mediated by Cu-containing proteins is scarce in the literature. Cu, Zn-superoxide dismutase (Cu, Zn-SOD), primarily known for dismutating superoxide radicals, has been shown to remove ONOO^−^ at rates of 9.4 × 10^3^ M^−1^ s^−1^, resulting in the nitration of tyrosine residues.^15^ However, Cu, Zn-SOD is consequently inactivated by ONOO^−^.^16^ Ceruloplasmin, a multicopper oxidase, has been shown to react with ONOO^−^, leading to copper release and loss of ferroxidase activity.^17^ Although the literature on Cu-containing proteins and their reactivity with ONOO^−^ is limited, these findings highlight their potential importance in cellular defense mechanisms against oxidative and nitrosative stress. Further research is needed to fully understand the scope and significance of copper proteins in ONOO^−^ reduction.

Amyloid precursor protein (APP) is a transmembrane protein implicated in the pathogenesis of Alzheimer's disease through its proteolytic processing, which generates amyloid-β (Aβ) peptides that aggregate into amyloid plaques.^18,19^ Among the various domains of APP, the E2 domain is particularly noteworthy due to its role in copper coordination. The E2 domain, located within the large extracellular region of APP, binds copper ions with a dissociation constant of *K*_D_ ∼ 10^−12^ M.^20^ This domain has been proposed to play roles in iron homeostasis, copper trafficking, and oxidative stress response.^20–22^

Recently, we investigated the Cu-binding site of the E2 domain using EXAFS, UV-Vis, and EPR spectroscopy, and discovered a previously unrecognized labile water ligand coordinating to the Cu(ii) cofactor, in addition to the four known histidines.^22^ The presence of this fifth water ligand in the first coordination sphere of Cu(ii)-E2, along with the positively charged protein surface, suggests potential reactivity with small anionic substrates like ONOO^−^. Indeed, Cu(i)-E2 reduces ONOO^−^ at a rate of 1.1 × 10^5^ M^−1^ s^−1^.^22^ The ability of E2 to remove peroxynitrite from solution is comparable to that of native peroxynitrite scavengers like Prxs and is rapid compared to other metalloproteins. Most importantly, the protein endures multiple redox cycles with peroxynitrite without damage to its active site.

Understanding the mechanisms of ONOO^−^ reduction by the E2 domain of APP is crucial not only for expanding the scope and significance of copper proteins in ONOO^−^ reduction but also for elucidating their physiological and pathological roles, particularly in the context of Alzheimer's disease. In this study, we elucidate the mechanism of ONOO^−^ reduction by Cu(i)-E2 by determining the reaction products using the Griess assay. Our results showed that equal amounts of nitrite and nitrate were formed, indicating that Cu(i)-E2 reduces ONOO^−^*via* single electron transfer (SET) to form NO_2_, which then undergoes disproportionation into nitrite and nitrate. This finding aligns with the EPR results, which demonstrated that Cu(i)-E2 is oxidized to Cu(ii)-E2 upon ONOO^−^ reduction. Furthermore, we investigated the modulation of ONOO^−^ reduction by manipulating secondary sphere interactions. A series of mutations at Lys435 were introduced to assess the role of the secondary coordination sphere in substrate recruitment. The ONOO^−^ reduction rates of these mutants were measured using stopped-flow techniques. As predicted, the K435E, K435L, and K435Q mutants exhibited slower reduction rates than the wild type due to electrostatic effects. However, unexpectedly, the K435R mutant also showed a slower rate. These anomalies suggest that factors beyond electrostatics must be considered in substrate recruitment. Further exploration using MD simulations indicated that steric blocking introduced by a newly formed hydrogen bond contributes to this slower rate.

## Experimental

### General

A MilliQ IQ 7000 Lab Water System (resistivity = 18.2 MΩ cm) was used for water purification.

### Chemicals

GelCode blue stain reagent, GeneRuler 1 kb Plus DNA ladder, and Ni-NTA (nitriloacetic acid) Superflow agarose were purchased from Thermo Scientific. ACS grade copper(ii) sulfate pentahydrate, PCR Master Mix (K0172), and α-toluenesulfonyl fluoride (PMSF) were purchased from Alfa Aesar. ACS-grade sodium chloride, imidazole, tryptone, yeast extract, ACS-grade l-ascorbic acid, ACS-grade sodium nitrite, 30% hydrogen peroxide, ACS-grade sodium hydroxide, and Tris base were purchased from Fisher Chemical. Manganese(iv) oxide was purchased from Millipore Sigma. Kanamycin monosulfate and isopropyl-β-d-thiogalactoside (IPTG) were purchased from Goldbio.

### PCR and site-directed mutagenesis

The E2 domain of amyloid precursor protein (APP), residues 295–500 of APP_695_, was synthesized and cloned into a pET-47b(+) vector using the kflI and *Bam*HI restriction sites. The plasmid construct was confirmed *via* Sanger sequencing. To achieve the desired mutations (K435E, K435L, K435Q, K435R), PCR was performed using the necessary forward (F) and reverse (R) primers, PCR Master Mix (K0172) reagents, and a BioRad C1000 Touch Thermal Cycler. Plasmids containing the mutation at K435 were transformed and amplified in NEB Turbo competent cells. Successful mutagenesis was confirmed *via* Sanger sequencing and cell stocks containing proper mutations were stored at −80 °C and freshly streaked before use. Plasmids were stored at −20 °C. The primers utilized above, written in 5′ to 3′ orientation, are as follows: K435E forward primer – TACCCTGGAACATTITGAACATGTTCG TATGGTGG, K435E reverse primer – AAATGTTCCAGGGTATGCT GACGATCTTCTG, K435L forward primer – TACCCTGCTGCATTT TGAACATGTTCGTATGGTGG, K435L reverse primer – AAATGCA GCAGGGTATGCTGACGATCTTTCTG, K435Q forward primer – TA CCCTGCAGCATTTTGAACATGTTCGTATGGTGG, K435Q reverse primer – AAATGCTGCAGGGTATGCTGACGATCTTTCTG, K435R forward primer – TACCCTGCGTCATTTTGAACATGTTCGTATGGT GG and K435R reverse primer – AAATGACGCAGGGTATGCTG ACGATCTTTCTG.

### Mutant protein expression and purification

Plasmids containing the desired mutations were transformed into BL21(DE3) competent cells. The transformed cells were grown at 37 °C in Luria–Bertani (LB) medium with 50 μg mL^−1^ kanamycin to an OD_600_ = 0.8. Cell cultures were induced with 0.6 mM IPTG for 4 hours, harvested, and resuspended in lysis buffer (25 mM Tris, 150 mM NaCl, 100 μM PMSF, pH = 7.4). Cells were later sonicated on ice using 10 s pulses followed by 30 s of rest and cellular debris were pelleted *via* centrifugation. Immobilized metal ion affinity chromatography was employed to purify E2 mutants from solution. 6x Histidine tags were removed by incubating E2 mutants in a 1 : 100 mole ratio with human rhinovirus (HRV) 3C for 4 hours at 4 °C.^23^ Ni-NTA affinity chromatography was used to separate HRV-3C and the cleaved 6xHis tag from the mutant of interest. E2 mutants were further purified *via* a HiPrep 16/60 Sephacryl S-100 HR size exclusion column on a BioRad NGC chromatography system. The concentration of wild-type and mutant apo-E2 was estimated *via* UV-Vis absorbance at 280 nm using an extinction coefficient of 14 440 M^−1^ cm^−1^.^22^

### Reconstitution of apo-E2 mutants

E2 mutant proteins were incubated with 2 mole equivalents of Cu(ii) sulfate pentahydrate under stirring for 2 hours at 4 °C. Unbound Cu(ii) was removed *via* a PD10 desalting column. Following copper reconstitution, Cu(ii)-E2 mutants were used directly. When Cu(i)-E2 mutants were required, the metal cofactor from Cu(ii)-E2 mutants was reduced to Cu(i) by titrating 5 mole equivalents of l-ascorbic acid into solution under stirring for 30 minutes at 4 °C under a 98% N_2_ and 2% H_2_ atmosphere in a COY Type B vinyl anaerobic chamber with the oxygen content monitored using a CAM-12 monitor. Excess sodium ascorbate was removed *via* a PD10 desalting column.

### HRV-3C protein expression and purification

The pET-47b(+) plasmid containing the HRV-3C gene was purchased from Addgene (Plasmid #162795). HRV-3C was expressed and purified following previously published methods.^24^ Briefly, the pET-47b(+) plasmid was transformed into *E. coli* BL21(DE3) cells, and the culture was grown in LB medium containing 50 mg L^−1^ kanamycin at 37 °C to an OD_600_ of 0.8. Upon reaching the desired cell density, the temperature was reduced to 14 °C, and protein expression was induced by adding 0.8 mM IPTG, followed by incubation for 18 hours. Cells were harvested and lysed *via* sonication. HRV-3C was purified *via* Ni-NTA affinity chromatography followed by a HiPrep 16/60 Sephacryl S-100 HR size exclusion column. The purified protein was diluted to include a final concentration of 10% glycerol v/v and stored at −80 °C. HRV-3C aliquots were thawed just prior to use.

### Copper quantification

Cu(i)-E2 mutants were mixed in a 1 : 1 ratio with 500 mg L^−1^ biquinoline dissolved in glacial acetic acid, along with an excess of cysteine added to the solution.^25^ After 1 hour of incubation, samples were analyzed *via* UV-Vis spectroscopy. The copper concentration was calculated from absorbance values at 547 nm, quantified with an experimentally determined extinction coefficient of 7300 M^−1^ cm^−1^ (internal standard in our working buffer) and compared to the calculated concentration of the protein (Fig. S5†).

### Circular dichroism

Cu(ii)-E2 mutants were exchanged into a 25 mM Tris, 50 mM Na_2_SO_4_, pH = 7.4 buffer to assess the secondary structure. CD measurements were collected on a JASCO J-1700 circular dichroism spectrophotometer and spectra were collected under the following conditions: scan rate 50 nm min^−1^, scan number 2, temperature 21.1 °C, and concentration 7 μM.

### EPR characterization

EPR spectra were recorded on a Bruker EMXplus EPR equipped with a ColdEdge Stinger closed-cycle flow system. Cu(ii)-E2 and Cu(i)-E2 samples were prepared as described above. Except when specified, the EPR samples contained approximately 350 μM E2 mutants in 25 mM Tris Buffer (pH = 7.4) with 150 mM NaCl. All samples were flash frozen and stored in liquid nitrogen before analysis. EPR spectra were recorded with the following conditions: temperature 20 K, modulation amplitude 4 G, microwave power 5.0 mW, and microwave frequency ∼9.5 GHz. The EPR spectra were simulated using EasySpin.^26^

### Synthesis of peroxynitrite

Peroxynitrite was synthesized using a Masterflex™ L/S™ Variable-Speed Console Drive by mixing hydrogen peroxide and nitrite under acidic conditions for 0.37 s before being quenched in a basic solution as previously described.^22,27^ Excess hydrogen peroxide was eliminated from the solution by incubation with MnO_2_ for 15 minutes. Vacuum filtration was utilized to remove MnO_2_. The peroxynitrite concentration was determined *via* UV-Vis spectroscopy at 302 nm (*ε* = 1700 M^−1^ cm^−1^).^27^

### Kinetics of single turnover peroxynitrite scavenging activity

The single turnover peroxynitrite scavenging abilities of Cu(i)-E2 mutants were assayed at 20.0 °C as previously described.^22,28^ In brief, 60, 80, 100 or 120 μM Cu(i)-E2 mutants in a 25 mM Tris and 150 mM NaCl buffer at pH 7.4 were mixed with 30 μM peroxynitrite in 5 mM NaOH in a 1 : 1 ratio. It was ensured that the reaction mixture was maintained at pH = 7.4 post mixing. The reduction of peroxynitrite was monitored by the decrease in absorption at 302 nm using an Applied Photophysics SX-20 stopped flow system with a PDA detector. A minimum of 3 spectra were obtained and the non-linear fitting was applied using *y* = *y*_0_ + *A*_1_*e*^−(*x*−*x*_0_)/*t*_1_^, where *y* is the absorbance at 302 nm and *x* is the time, and 1/*t*_1_ is the rate constant. These pseudo-first-order rate constants were then plotted as a function of mutant concentration to calculate the second-order rate constant for each mutant.

### Determination of the products of protein mediated peroxynitrite reduction

The products of protein mediated peroxynitrite reduction were detected *via* a Cayman Chemical Nitrate/Nitrite Colorimetric Assay Kit. Cu(i)-E2 was incubated with one equivalent of peroxynitrite for 2 minutes with stirring under a 98% N_2_ and 2% H_2_ atmosphere. This solution was brought out of the anaerobic atmosphere and was incubated with either buffer or nitrate reductase and its cofactors for one hour. The sample was then mixed with Griess reagents for 10 minutes to convert nitrite into a purple azo compound. Quantification of this compound was performed using absorbance at 540 nm. Molar absorptivity was calculated using a standard curve using known nitrite concentrations. Nitrite produced in the experiment was determined using the samples lacking the addition of nitrate reductase and its cofactors. To determine nitrate production, the same sample was prepared in the presence of nitrate reductase and cofactors. The amount of nitrite produced in this experiment was subtracted from samples lacking nitrate reductase to determine the nitrate concentration.

### Molecular dynamics simulation

The Cu coordination was parameterized using DFT calculations with the open-source program MCPB.py from AmberTools.^29^ MCPB.py employed two model systems to balance accuracy and computational efficiency. The smaller model system was used to determine bond and angle parameters associated with the metal, with force constants derived using the Seminario method from the Hessian matrix obtained in DFT calculations. The larger model system was utilized for parameterizing partial charges through Merz-Singh-Kollman RESP calculations.^30^ Once the Cu coordination parameters were generated, molecular dynamics (MD) simulations were conducted using the Amber simulation package^31^ for both the wild-type protein (PDB: 3UMK) and all mutants. All simulations were performed within an orthorhombic simulation box with dimensions of 117.2 Å × 76.1 Å × 64.0 Å, containing 14 458 explicit OPC water molecules^32^ with periodic boundary conditions, using the Amber force field ff19SB.^33^ Long-range electrostatic interactions were calculated using the Particle Mesh Ewald (PME) method, with an 8.0 Å real-space cutoff for both van der Waals and electrostatic interactions. The SHAKE algorithm^34^ was applied to constrain all bond lengths involving hydrogen atoms. The structures of all systems were first minimized using a combination of steepest descent and conjugate gradient algorithms to eliminate initial steric clashes and unfavorable interactions. After structural minimization, the systems were equilibrated using 5 ns NPT simulations at 300 K and 1 bar. Temperature was controlled with a Langevin thermostat,^35^ while pressure was regulated using isotropic pressure scaling.^36^ The Verlet algorithm, with a time step of 1.0 fs, was used to integrate the equations of motion. Following equilibration, 250 ns production NPT simulations were performed for the wild-type protein and all K435 mutants, using the same simulation parameters as those in the equilibration phase.

## Results

### Elucidating the mechanism of ONOO^−^ reduction by Cu(i)-E2 through reaction product analysis using the Griess assay

**Fig. 1:**
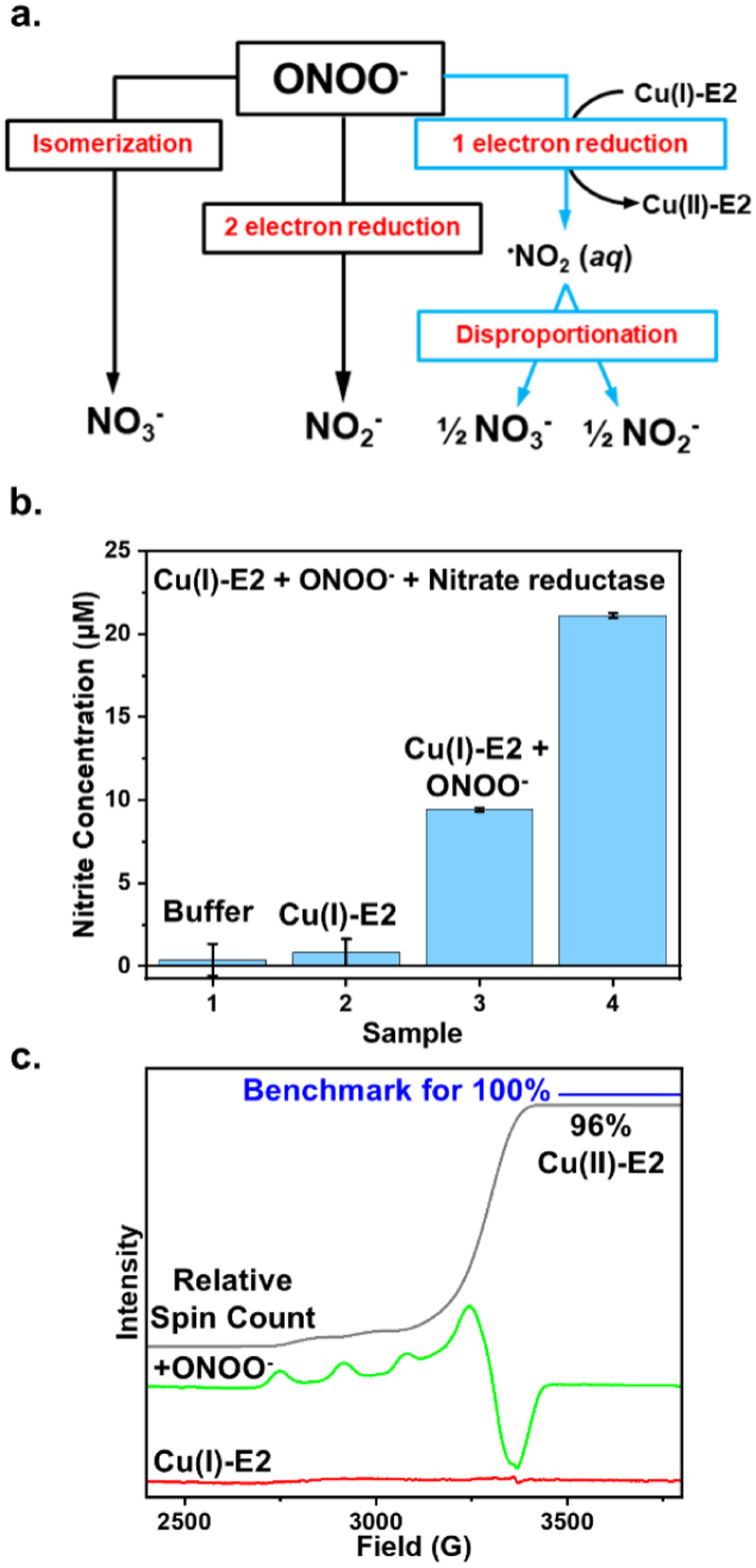
Mechanism of ONOO^−^ reduction by Cu(i)-E2 analyzed through reaction products using the Griess assay. (a) The three potential ONOO^−^ removal pathways mediated by enzymes. (b) The Griess assay was used to quantify nitrite formation when Cu(i)-E2 reacts with ONOO^−^, both in the presence and absence of nitrate reductase, with the reaction buffer serving as the control. The amount of nitrate formed was determined by subtracting the nitrite measured in the absence of nitrate reductase from that in the presence of nitrate reductase. The reaction was repeated three times to calculate the error bars. (c) EPR spectra of Cu(i)-E2 reacting with ONOO^−^ under anaerobic conditions show that Cu(ii)-E2 is recovered with a 96% yield, based on the total spin count of the sample compared to a Cu(ii) standard.

The removal of ONOO^−^ from solution by enzymes can occur through three primary pathways: (1) isomerization to exclusively form nitrate, (2) two-electron reduction to exclusively form nitrite, or (3) one-electron reduction to form NO_2_, which then undergoes disproportionation in water, leading to the formation of both nitrite and nitrate (Fig. 1a). For example, heme proteins can coordinate to peroxynitrite and isomerize it into the less harmful nitrate anion.^4^ Prxs catalyze the two-electron reduction of ONOO^−^ to form nitrite exclusively.^37^ In contrast, the one-electron reduction of peroxynitrite generates NO_2_, which can either disproportionate to form both nitrate and nitrite in aqueous solution^38,39^ or nitrify tyrosine residues, as observed in Cu, Zn-SOD.^37^ Therefore, by determining the products of ONOO^−^ reduction, we can elucidate the mechanism and pathway through which Cu(i)-E2 interacts with ONOO^−^.

The Griess reagent converts nitrite into a purple azo compound, allowing for the quantification of nitrite based on absorbance at 540 nm. As shown in Fig. 1b, both the buffer control and Cu(i)-E2 alone showed no detectable nitrite. In the experiment, 20 μM Cu(i)-E2 was incubated with one equivalent of ONOO^−^ under anaerobic conditions inside a glovebag. After 2 minutes, the solution was mixed with the Griess reagents for 10 minutes, resulting in the detection of 9.4 μM nitrite (Fig. 1b). To quantify nitrate, nitrate reductase is added to quantitatively convert nitrate into nitrite. The total nitrite in the solution is then measured using the Griess assay. By subtracting the nitrite measured without nitrate reductase from the nitrite measured with it, the amount of nitrate in the solution can be determined. The Griess assay demonstrates near-equimolar amounts of nitrite (9.4 μM) and nitrate (11.6 μM) formed in solution (Fig. 1b), suggesting that Cu(i)-E2 undergoes a SET to ONOO^−^, reducing it to NO_2_, which subsequently undergoes disproportionation in solution. These results are further supported by EPR studies, which detected a quantitative amount of Cu(ii)-E2 when Cu(i)-E2 was treated with ONOO^−^ (Fig. 1c).^22^

### Spectroscopic characterization of Cu(ii)-E2 mutants

In metalloenzymes, secondary sphere interactions can modulate redox potential,^40^ guide substrates electrostatically to the active site,^41^ influence reaction mechanisms,^42^ and create steric clashes^43^ – all of which can affect enzymatic activity. The predicted surface charge density map of Cu(ii)-E2 indicates a positive electrostatic potential surface around the substrate-access funnel (Fig. S1†). A positively charged residue, Lys435, is located 6.3 Å away from the Cu site in E2. Since the substrate ONOO^−^ is negatively charged, we hypothesize that this electrostatic interaction plays a crucial role in ONOO^−^ reduction activity. To test this hypothesis, a series of mutants—K435E, K435L, K435Q, and K435R—were created to introduce negative, neutral, less polar, or positive charges. Examining this electrostatic interaction within the secondary coordination sphere helps us understand how nature designs enzymes for ONOO^−^ scavenging.

Site-directed mutagenesis using PCR was employed to introduce the desired mutations into the E2 domain of APP. The mutations were confirmed by Sanger sequencing. Plasmids containing the confirmed mutations were then transformed into BL21(DE3) *E. coli* cells. Each mutant was expressed and purified using previously reported methods,^22^ and its purity was verified *via* SDS-PAGE (Fig. S2†). The holo protein was reconstituted by incubating the apo mutant with two equivalents of CuSO_4_ in a buffer. Excess copper salt was removed by passing the mixture through a PD-10 desalting column. To quantify the Cu/protein ratio, the protein concentration was determined by its absorption at 280 nm, and the copper content was quantified using the biquinoline assay. In this assay, Cu(ii) in the sample is reduced to Cu(i), which then binds to the biquinoline reagent to form a pink-colored complex with an absorption peak at 574 nm (Fig. S3–S5†). Our results show a 1 : 1 Cu/protein ratio, indicating that each of the tested E2 mutants exhibits stoichiometric coordination of Cu(ii) (Fig. S5†).

**Fig. 2:**
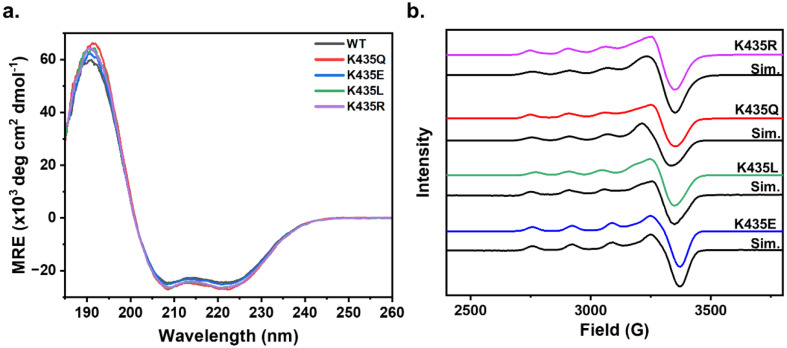
Spectroscopic characterization of Cu(ii)-E2 mutants. (a) CD spectra of Cu(ii)-E2 mutants K435R (pink), K435Q (red), K435L (green), and K435E (blue) in 25 mM Tris/50 mM Na_2_SO_4_ at pH 7.4. (b) EPR spectra of Cu(ii)-E2 mutants K435R (pink), K435Q (red), K435L (green), and K435E (blue) along with their respective simulations (black) in 25 mM Tris, 150 mM NaCl, and pH 7.4. EPR conditions: temperature 20 K, modulation amplitude 4 G, microwave power 5.0 mW, and microwave frequency ∼9.47 GHz.

Upon confirming the stoichiometric incorporation of Cu(ii) by the E2 mutants, we sought to determine whether the secondary sphere mutations led to any changes in the overall folding of the E2 domain. Circular dichroism (CD) spectroscopy is a valuable tool for assessing protein secondary structure. Proteins with a high α-helical content typically display a positive absorbance band at 193 nm and two negative features at 208 nm and 222 nm, while proteins with the β-sheet character exhibit a positive band at 195 nm and a negative band at 218 nm.^44^ The CD spectra of wild-type (WT) Cu(ii)-E2 show a prominent positive feature at 193 nm and two negative features at 208 nm and 222 nm, consistent with the highly α-helical nature of the protein.^20,45^ The CD spectra of the Cu(ii)-E2 mutants overlap with the WT (Fig. 2a), indicating that the secondary structure is conserved despite these mutations.

To determine whether the mutants induce any electronic changes at the Cu active site, we continued our investigation of the electronic structure of Cu(ii)-E2 mutants using continuous wave electron paramagnetic resonance (EPR) spectroscopy. The EPR spectra show that in all mutants, *g*_*z*_ > *g*_*x*_ ≈ *g*_*y*_, with *A*_*z*_ values typical of a mononuclear type 2 Cu center featuring a d_*x*^2^−*y*^2^_ ground state (Fig. 2b). The minimal deviations in *g* and *A*_*z*_ values across the mutants suggest that the secondary sphere mutations do not significantly alter the electronic structure of the Cu centers (Table 1). Subsequently, the Cu(ii)-E2 sample was anaerobically reduced inside a glovebox using 5 molar equivalents of sodium ascorbate, with the excess ascorbate subsequently removed *via* a PD10 desalting column. The resulting sample was EPR silent (Fig. S6†), indicating the complete reduction of Cu(ii) to Cu(i) in the E2 mutants.

**Table 1:** EPR simulation parameters for Cu(ii)-E2 mutants

Mutation	*g*-Values (*g*_‖_, *g*_⊥_)	*A*_‖_ (× 10^−4^ cm^−1^)
K435R	2.272, 2.050	160
K435Q	2.270, 2.050	163
K435L	2.280, 2.050	163
K435E	2.262, 2.060	164

### Modulating ONOO^−^ reduction activity through secondary coordination sphere editing

**Fig. 3:**
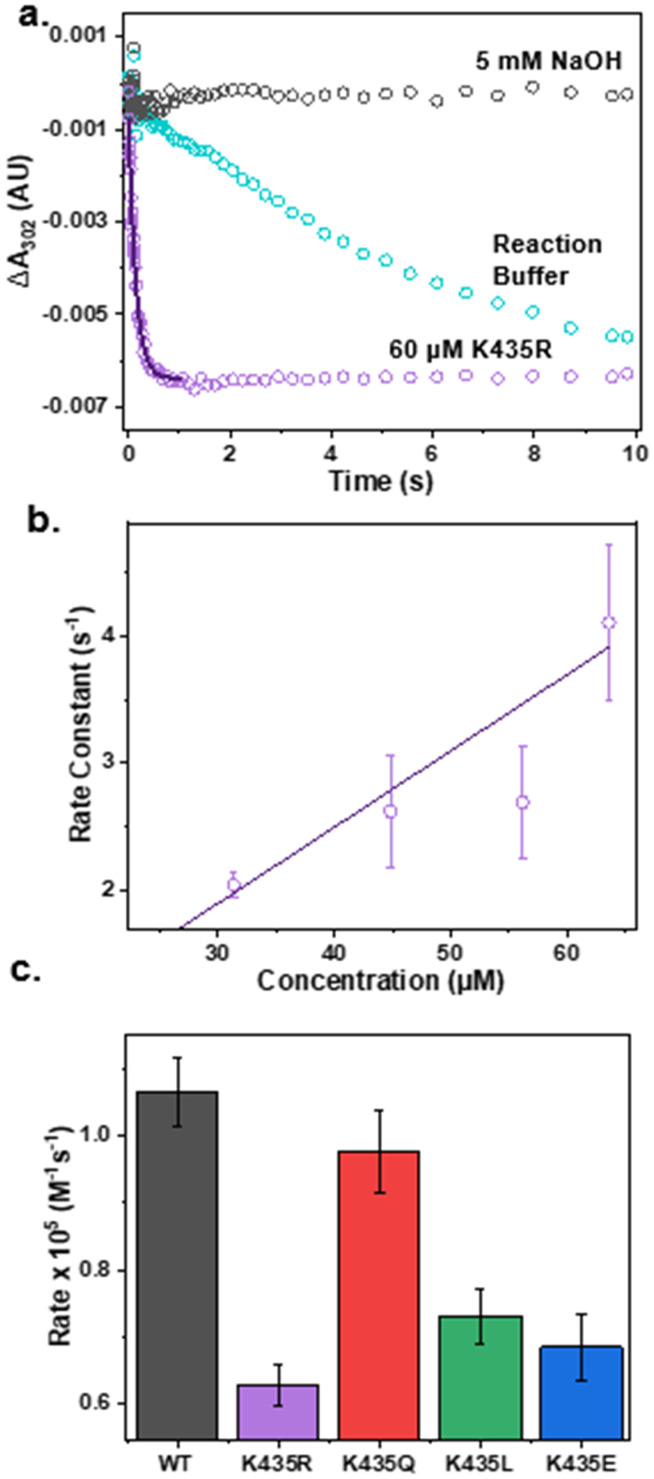
Removal of ONOO^−^ by Cu(i)-E2 mutants. (a) The reduction of peroxynitrite was monitored by the decrease in absorption at 302 nm using stopped-flow spectroscopy in 5 mM NaOH (gray), reaction buffer (blue), and in the presence of the Cu(i)-E2 K435R mutant in the reaction buffer (purple) with its respective linear fit (black). (b) Pseudo-first-order rate constants for peroxynitrite reduction as a function of K435R concentration. (c) Second order reaction rates for ONOO^−^ removal by Cu(i)-E2 and mutants.

After confirming that the mutants have minimal impact on the secondary structure of the E2 domain and the electronic structure of the active site, we proceeded to test our hypothesis that electrostatic interactions in the secondary coordination sphere play a crucial role in ONOO^−^ reduction activity. The pseudo-first-order reaction rate of each mutant for ONOO^−^ reduction was measured by anaerobically mixing ONOO^−^ with an excess amount of Cu(i)-E2 mutants in a stopped-flow spectrometer. The reduction of ONOO^−^ was monitored by the decrease in absorbance at 302 nm. As shown in Fig. 3a, ONOO^−^ undergoes slow self-decay over several seconds in the reaction buffer. However, in the presence of the Cu(i)-E2 K435R mutant, ONOO^−^ reduction was completed within 400 ms. Non-linear fitting was applied to the first 400 ms to determine the pseudo-first-order reaction rate. The second-order rate constant for peroxynitrite reduction was determined by plotting the pseudo-first-order rate constant as a function of Cu(i)-E2 concentration (Fig. 3a). As expected, less polar and neutral mutants, such as K435Q and K435L, exhibited slower rates (0.97 and 0.73 × 10^5^ M^−1^ s^−1^, respectively) compared to the wild-type (1.1 × 10^5^ M^−1^ s^−1^) (Fig. S7–10† and 3c). Furthermore, the negatively charged K435E mutant exhibited an even slower rate (0.68 × 10^5^ M^−1^ s^−1^) compared to K435Q and K435L. Much of the observed variation in rates can be attributed to simple electrostatics. However, contrary to expectations, the K435R mutant, which has a positively charged side chain, reduced peroxynitrite at the slowest rate among all the mutants, with a rate of 0.63 × 10^5^ M^−1^ s^−1^.

### Using molecular dynamics simulations to rationalize ONOO^−^ reduction activity modulated by secondary coordination sphere editing

To investigate why the K435R mutant exhibits the lowest rate of ONOO^−^ removal compared to the native protein and other mutants, we performed molecular dynamics (MD) simulations on WT and all K435 mutants. We first parameterized the four-histidine-coordinated Cu active site using density functional theory (DFT). To achieve a balance between accuracy and computational efficiency, we employed two model systems for parameterization (Fig. S11†). The smaller model system was used to determine bond and angle parameters associated with Cu, with force constants derived using the Seminario method from the Hessian matrix obtained in DFT calculations. The larger model system was used for parameterizing partial charges through Merz-Singh-Kollman RESP calculations.^30^

**Fig. 4:**
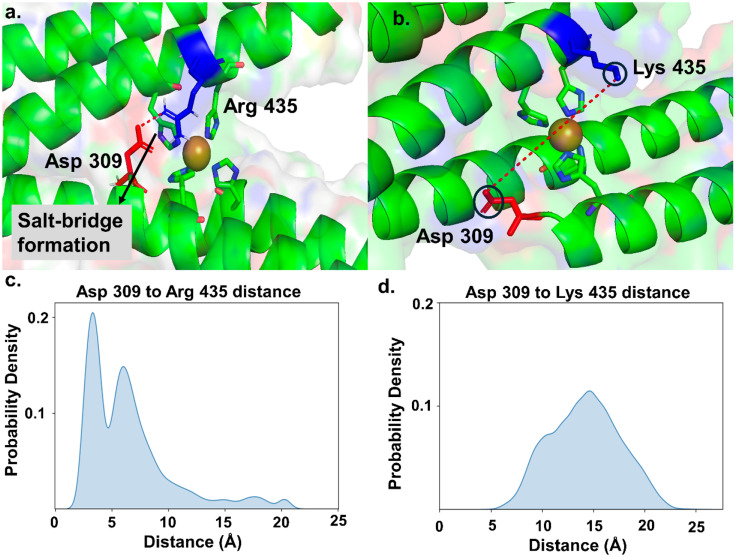
Molecular dynamics simulations of K435R and WT. (a) The side chain of K435R electrostatically interacts with the carboxylate group of D309. The probability density analysis of the distance, shown in (c), peaks around 4 Å. (b) In WT, the side chain of K435 remains in the solvent, rotating freely. The probability density analysis of the distance, shown in (d), extends over a wide range.

Once these parameters were generated, we proceeded with the simulations using the Amber simulation package^31^ for the WT protein and all K435 mutants. We performed salt bridge analysis (using VMD)^46^ and hydrogen bond analysis on the side chains of the WT protein and mutants to examine their orientation. In the WT protein, we did not observe salt bridge or hydrogen bond formation for K435. As a result, the side chain of K435 moved freely, allowing the diffusion of ONOO^−^ to the Cu center (Fig. 4b). In contrast, in the K435R mutant, the side chain of K435R electrostatically interacts with the carboxylate group of D309, forming a salt bridge (Fig. 4a). This observation is supported by the probability density analysis of the salt bridge distance in Fig. 4c, which shows that the probability for the K435R mutant peaked at 4 Å. In comparison, the probability distribution for WT K435 extends over a wide range (Fig. 4d), with K435 and D309 remaining separated by 14–15 Å throughout the simulation. Consequently, the K435 side chain remains in the solvent, rotating freely. Therefore, we concluded that the salt bridge between K435R and D309 restricts the side chain of K435R to a specific orientation over the Cu active site, thereby blocking the diffusion of negatively charged ONOO^−^ to the active site. Similarly, we analyzed two other mutants, K435L and K435E, to determine if their side chains are involved in any interactions. We found that they do not participate in salt bridge or hydrogen bond formation (Fig. S12†). In both mutants, the side chains of K435L and K435E rotate freely, surrounded by water molecules, which allows the diffusion of peroxynitrite to the active site. The slower ONOO^−^ reduction rates observed for K435L and K435E are primarily due to electrostatic factors.

## Discussion

Electrostatic effects play a crucial role in metalloprotein catalysis, significantly influencing the reactivity, selectivity, and efficiency of catalytic processes. For instance, in Cu, Zn-SOD, the copper ion only accounts for 0.1% of the protein's surface yet the protein can remove superoxide at rates near the diffusion controlled limit. This remarkable speed is attributed to positively charged residues surrounding the active site attracting the negatively charged superoxide anion, thereby increasing its local concentration near the catalytic center.^47^ This enhancement in substrate concentration directly contributes to the enzyme's catalytic efficiency. Similarly, in horseradish peroxidase, the electrostatic environment created by surrounding amino acid residues stabilizes the transition state during the breakdown of hydrogen peroxide,^48^ aiding in both substrate binding and proper orientation. In [NiFe] hydrogenase, charged amino acids near the active site facilitate proton-coupled electron transfer (PCET), which is essential for hydrogen oxidation, ensuring efficient proton transfer to and from the active site.^49,50^ These examples collectively underscore how electrostatic effects are integral to metalloprotein function, influencing key aspects such as substrate binding, transition state stabilization, and overall catalytic efficiency.

In addition to electrostatic effects, steric factors play a significant role in metalloprotein catalysis by influencing substrate binding and orientation within the active site. For example, in cytochrome P450, the shape of the substrate-binding pocket, defined by surrounding amino acids, determines the orientation and positioning of the substrate relative to the heme iron. This precise steric arrangement is crucial for controlling the site of oxidation on the substrate and, consequently, for determining the enzyme's substrate specificity and regioselectivity.^51^ In myoglobin and hemoglobin, the distal histidine within the heme pocket creates steric hindrance that controls the binding of oxygen and other small molecules, such as carbon monoxide (CO).^52^ This steric effect helps stabilize bound oxygen while preventing the strong binding of CO, which could otherwise outcompete oxygen. Site-directed mutagenesis of this histidine residue can lead to a 10- to 1000-fold increase in the rate of oxygen dissociation. Simultaneously, these mutations that decrease O_2_ affinity can increase CO affinity, illustrating how finely tuned metalloprotein active sites must be for proper function.^52^ Similarly, in nitric oxide synthase (NOS), steric effects and hydrogen bonds from surrounding residues in the active site, which houses a heme group and a tetrahydrobiopterin cofactor, control the positioning of l-arginine relative to the heme iron.^53^ These steric constraints are essential for the selective oxidation of l-arginine to nitric oxide (NO), ensuring the efficient and specific production of NO. These examples highlight how steric effects are critical for ensuring that substrates are properly positioned within the active site, that only specific reactions occur, and that enzymes maintain their catalytic efficiency and specificity.

Building on this understanding, our study highlighted the interplay between electrostatic and steric effects by creating a series of mutants—K435E, K435L, K435Q, and K435R—to introduce negative, neutral, less polar, or positive charges at a key position. As expected, the less polar and neutral mutants, K435Q and K435L, exhibited slower catalytic rates (0.97 and 0.73 × 10^5^ M^−1^ s^−1^, respectively) compared to the WT (1.1 × 10^5^ M^−1^ s^−1^). It is worth noting that the K435Q mutant is only slightly slower than the wild type. This can be attributed to the fact that the Gln side chain is still polar and flexible, allowing the amide group to interact with the peroxynitrite substrate *via* hydrogen bonding. The negatively charged K435E mutant showed an even slower rate (0.68 × 10^5^ M^−1^ s^−1^). However, the K435R mutant, which introduced a positively charged side chain, unexpectedly reduced peroxynitrite at the slowest rate among all the mutants, with a rate of 0.63 × 10^5^ M^−1^ s^−1^. This unexpected outcome results from the formation of a salt bridge between K435R and D309, which anchors the K435R side chain above the active site. This arrangement hinders the access of the negatively charged ONOO^−^ to the Cu ion (Fig. S13†). Our study underscores the crucial importance of balancing both electrostatic and steric effects in metalloprotein catalysis. The intricate interplay between these factors is fundamental to the design and function of metalloproteins. Achieving the right balance allows these proteins to catalyze reactions efficiently and selectively while maintaining their structural and functional integrity.

## Conclusions

In summary, we investigated the mechanism by which Cu(i)-E2 reduces ONOO^−^ by analyzing its reaction products using the Griess assay and EPR. Our results suggest that the reduction occurs *via* a SET process, where ONOO^−^ is reduced to NO_2_, which subsequently disproportionates into nitrite and nitrate in aqueous solutions. To explore the role of K435—a residue in the secondary sphere believed to be involved in substrate recruitment—in ONOO^−^ reduction activity, we generated a series of mutants: K435E, K435L, K435Q, and K435R, introducing negative, neutral, less polar, or positive charges, respectively. Investigating this electrostatic interaction within the secondary coordination sphere provides insight into how nature designs enzymes for ONOO^−^ scavenging. We first confirmed that these mutations did not significantly alter the secondary structure or the electronic structure of the Cu site, as demonstrated by CD and EPR analyses, respectively. The ONOO^−^ reduction activities of the mutants generally followed an electrostatic trend, which can be explained by the interaction of the negatively charged ONOO^−^ substrate with the charged residues, with K435R being the only exception. MD simulations revealed that the reduced activity of K435R can be attributed to the formation of a salt bridge between the mutated arginine residue and the nearby D309, which physically blocks ONOO^−^ from accessing the Cu active site. This study demonstrates how nature fine-tunes residues near the active site to facilitate the recruitment of small, negatively charged molecules. Our findings enhance the understanding of ONOO^−^ reduction by the E2 domain of APP, expand our knowledge of copper proteins in mitigating oxidative stress, and elucidate their physiological and pathological roles, particularly in Alzheimer's disease.

## Author contributions

E. C. Z., A. T. P. and S. T. conceived the present idea. E. C. Z, A. T. P., J. P., A. T., C. B. and J. L. carried out the experiment. S. T. provided resources and supervision for all experiments. D. P. and M. C. planned and carried out the simulations. A. T. P., E. C. Z., D. P., M. C. and S. T. wrote the original draft. All authors were involved in reviewing and editing.

## Conflicts of interest

There are no conflicts to declare.

## Supplementary Material

DT-054-D4DT02552K-s001

## Data Availability

The data supporting the findings of this study are available within the article and its ESI.† Any additional raw data generated during the study, including kinetic data, EPR spectra, and simulation files, are available from the corresponding author upon request.
