# Supplementary material for: Secondary sphere interactions modulate peroxynitrite scavenging by the E2 domain of amyloid precursor protein
Source: Dalton Trans. 2024 Dec 5;54(2):571–81. doi: 10.1039/d4dt02552k (PMC11640295; doi:10.1039/d4dt02552k)
Supplement: DT-054-D4DT02552K-s001 [file DT-054-D4DT02552K-s001.pdf]

Supplementary Information (SI) for Dalton Transactions.
This journal is © The Royal Society of Chemistry 2024

Supporting Information

Secondary Sphere Interactions Modulate Peroxynitrite Scavenging by the E2 Domain of Amyloid Precursor Protein

Eli C. Zuercher[1&], Andrew T. Poore[1&], Devendra Prajapat[1], Joseph Palazzo[1], Alana Thomas[1], Caitlin Birthright[1], Jack Lawrence[1], Ming Chen[1] & Shiliang Tian[1*]

[1]Department of Chemistry, Purdue University, West Lafayette, Indiana 47906, United States

*E-mail: sltian@purdue.edu

Table of Contents

	Page
**Fig. S1** Surface charge map of wild-type E2	S2
**Fig. S2** SDS-Page of mutant E2 samples	S2
**Fig. S3** Standard curve for Biquinoline assay in working buffer	S3
**Fig. S4** Biquinoline Assay of Mutant E2	S4
**Fig. S5 & S6** Cu(I)-E2 Mutant Cu incorporation and EPR spectra of Cu(I)-Mutants	S5
**Fig. S7-S10** Stopped-flow measurements for peroxynitrite decay by each mutant	S6-S9
**Fig. S11** Optimized Models for DFT	S10
**Fig. S12** MD simulations for K435E, K435L, and K435Q	S11
**Fig. S13** Surface map of wild-type Cu(II)-E2 compared to Cu(II)-K435R	S12

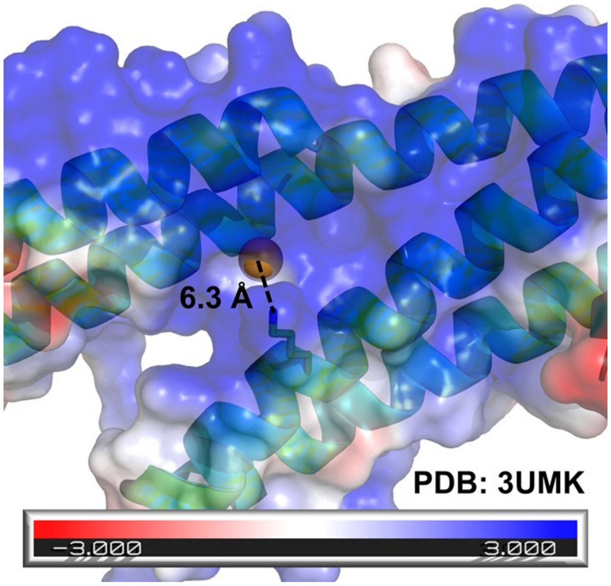

Figure S1. Surface charge map of wild type Cu(II)-E2. The solvent exposed Cu(I) center is located in a positively charged pocket which contains a nearby lysine residue only 6.3 Å away that could be utilized for substrate recruitment (PDB: 3UMK).

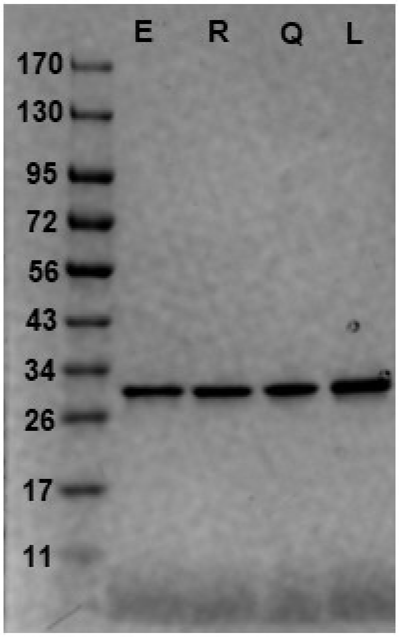

Figure S2. SDS-Page to demonstrate purity of E2 mutants K435E (left), K435R (middle left), K435Q (middle right), K435L (right). Samples were prepared utilizing 10 μM protein after protein purification and His-tag removal.

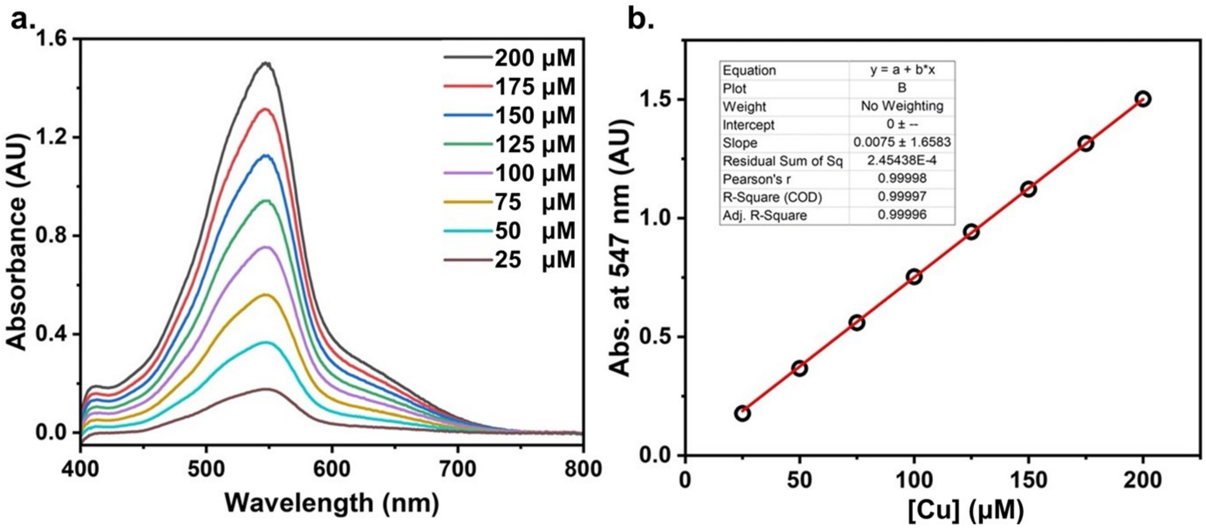

Figure S3. Biquinoline Colorimetric Copper Quantification Standard Curves. Samples containing known concentrations of $CuSO_4$ in solution were prepared in 25 mM Tris/150 mM NaCl at pH 7.4, and subjected to biquinoline (BQ) assay. a) UV-Vis absorbance measurements demonstrated a peak at 574 nm. b) Absorbance at 574 nm was plotted against the copper concentration, and a linear fit of the slope was used to calculate a molar extinction coefficient of 7.5 mM^{-1} cm^{-1} for the Cu(I)-BQ_2 complex in our buffer.

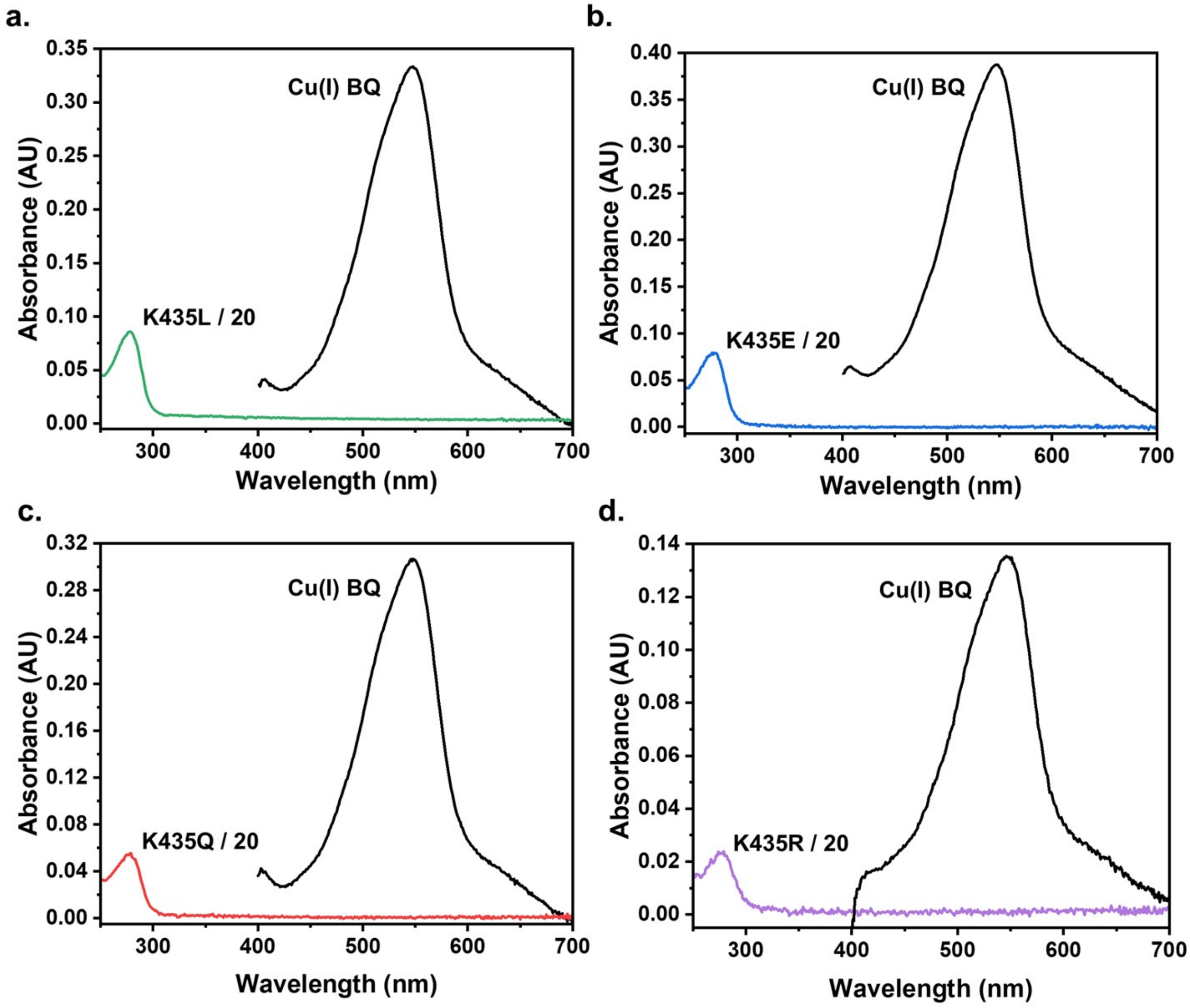

Figure S4. UV-Vis Absorbance of Biquinoline Samples. BQ samples were prepared with a 1:1 volume:volume ratio of holo-protein in 25 mM Tris, 150 mM NaCl, pH = 7.4 with a glacial acetic acid solution containing 500 mg Biquinoline per 1 L glacial acetic acid. Concentrations of mutant a. K435L b. K435E c. K435Q and d. K435R was determined prior to dilution with glacial acetic acid at 280 nm. Formation of Cu(I) biquinoline complex was measured at 574 nm.

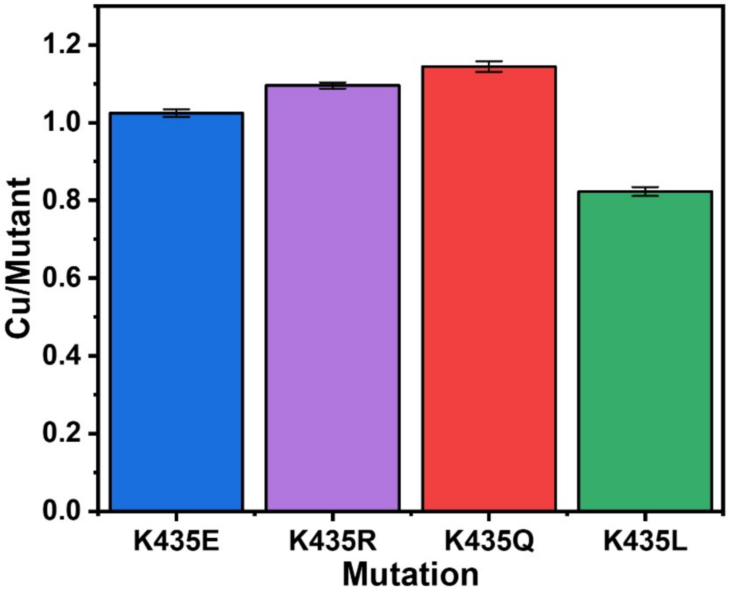

Figure S5. BQ Assay of the Cu(I)-E2 mutants. The incorporation of Cu into each mutant was calculated by utilizing the predicted molar extinction coefficient at 280 nm of each mutant with the empirically determined extinction coefficient for the Cu(I)-BQ_2 complex at 574 nm.

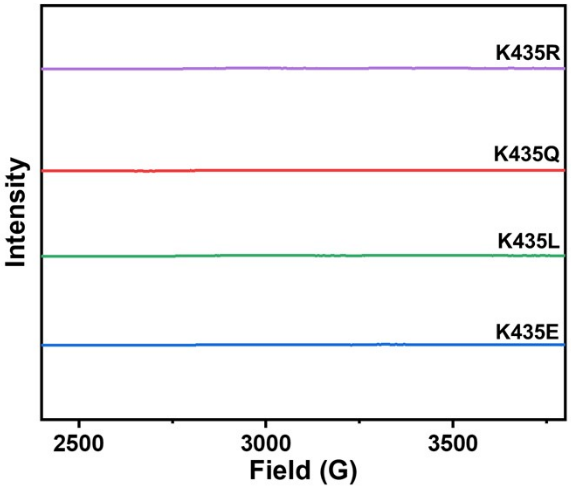

Figure S6. X-band EPR of the Cu(I)-E2 mutants. 350 μM Cu(I)-E2 mutants K435R (pink), K435Q (red), K435L (green), K435E (blue) were prepared and frozen anaerobically in 25 mM Tris, 150 mM NaCl, pH 7.4. The intensity is set to the same scale as figure containing the EPR of the Cu(II)-E2 mutants. Conditions: temperature 20 K, modulation amplitude 4 G, microwave power 5.0 mW, and microwave frequency ~9.47 GHz.

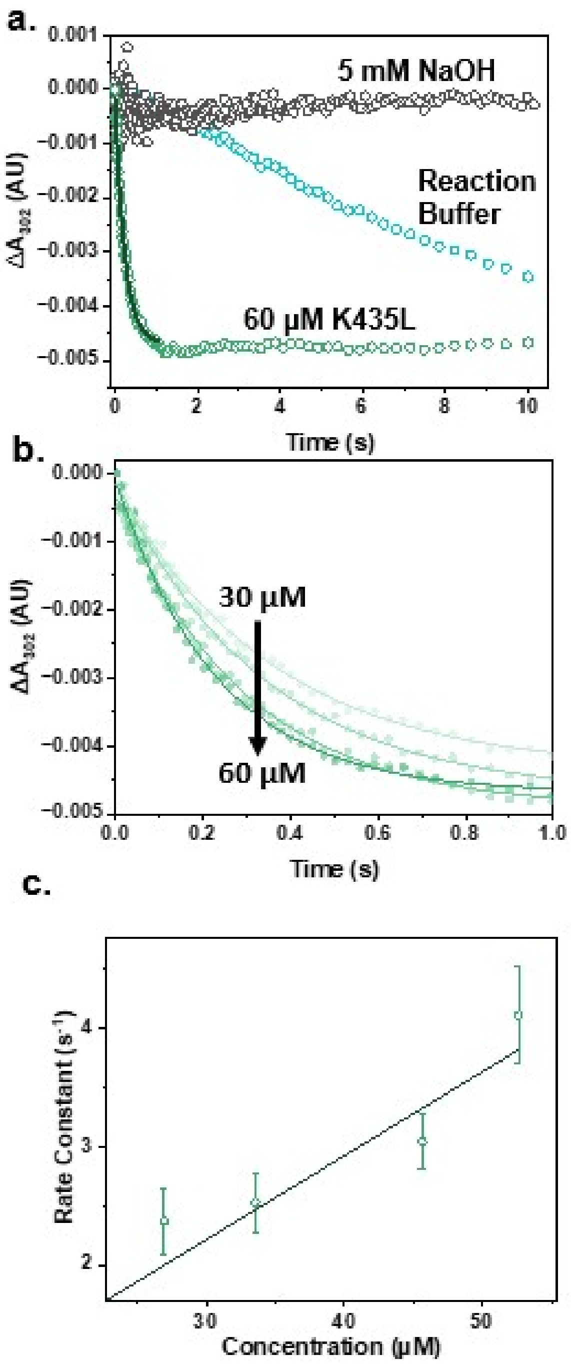

Figure S7. Peroxynitrite Decay of Mutant K435L. (a) Stopped flow spectra to follow the change in peroxynitrite concentration at 302 nm over time in the 5 mM NaOH (gray), reaction buffer (teal), and with 60 µM K435L in the reaction buffer (green). Non-linear fitting is applied to the first second of reactivity (black). (b) Change in absorbance at 302 nm in the first second of samples of K435L at various concentrations, with darker colors in the gradient green corresponding to higher concentrations of protein, and their respective fits. (c) Pseudo-first-order rate constants for peroxynitrite reduction as a function of K435L concentration.

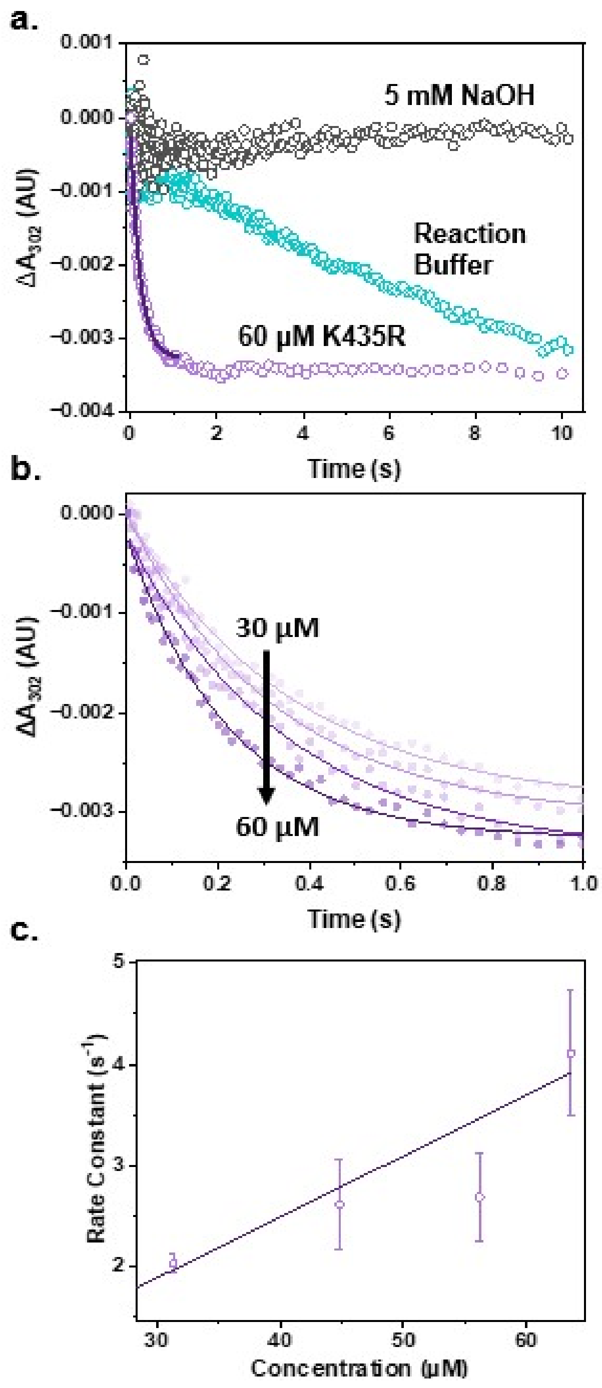

Figure S8. Peroxynitrite Decay of Mutant K435R. (a) Stopped flow spectra to follow the change in peroxynitrite concentration at 302 nm over time in the 5 mM NaOH (gray), reaction buffer (teal), and with 60 μM K435R in the reaction buffer (purple). Non-linear fitting is applied to the first second of reactivity (black). (b) Change in absorbance at 302 nm in the first second of samples of K435R at various concentrations, with darker colors in the gradient purple corresponding to higher concentrations of protein, and their respective fits. (c) Pseudo-first-order rate constants for peroxynitrite reduction as a function of K435R concentration.

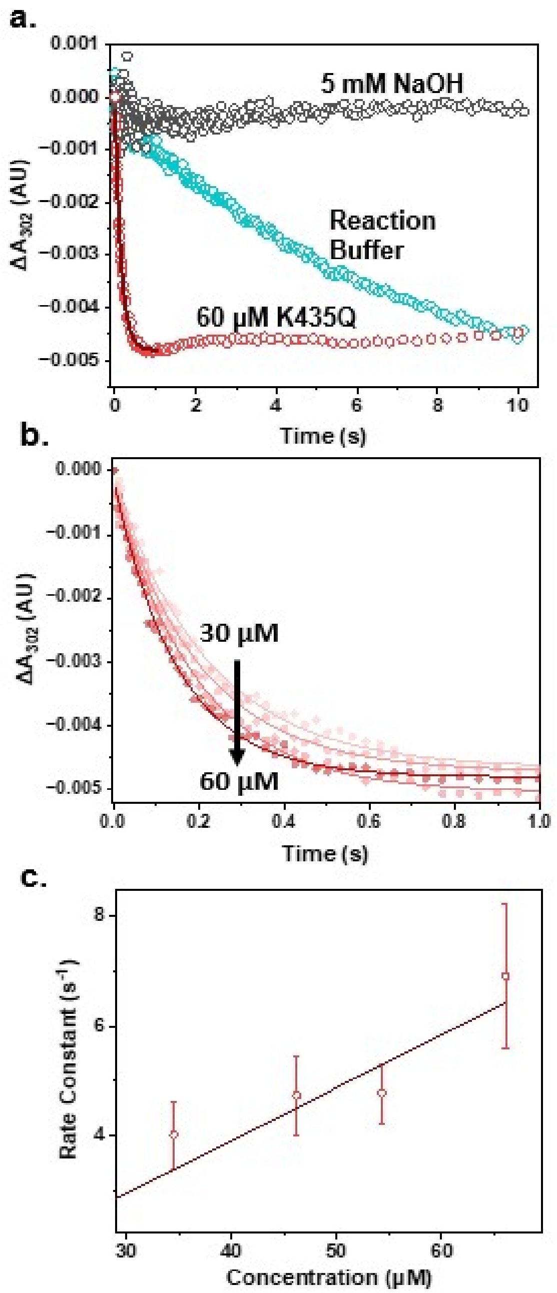

Figure S9. Peroxynitrite Decay of Mutant K435Q. (a) Stopped flow spectra to follow the change in peroxynitrite concentration at 302 nm over time in the 5 mM NaOH (gray), reaction buffer (teal), and with 60 μM K435Q in the reaction buffer (red). Non-linear fitting is applied to the first second of reactivity (black). (b) Change in absorbance at 302 nm in the first second of samples of K435Q at various concentrations, with darker colors in the gradient red corresponding to higher concentrations of protein, and their respective fits. (c) Pseudo-first-order rate constants for peroxynitrite reduction as a function of K435Q concentration.

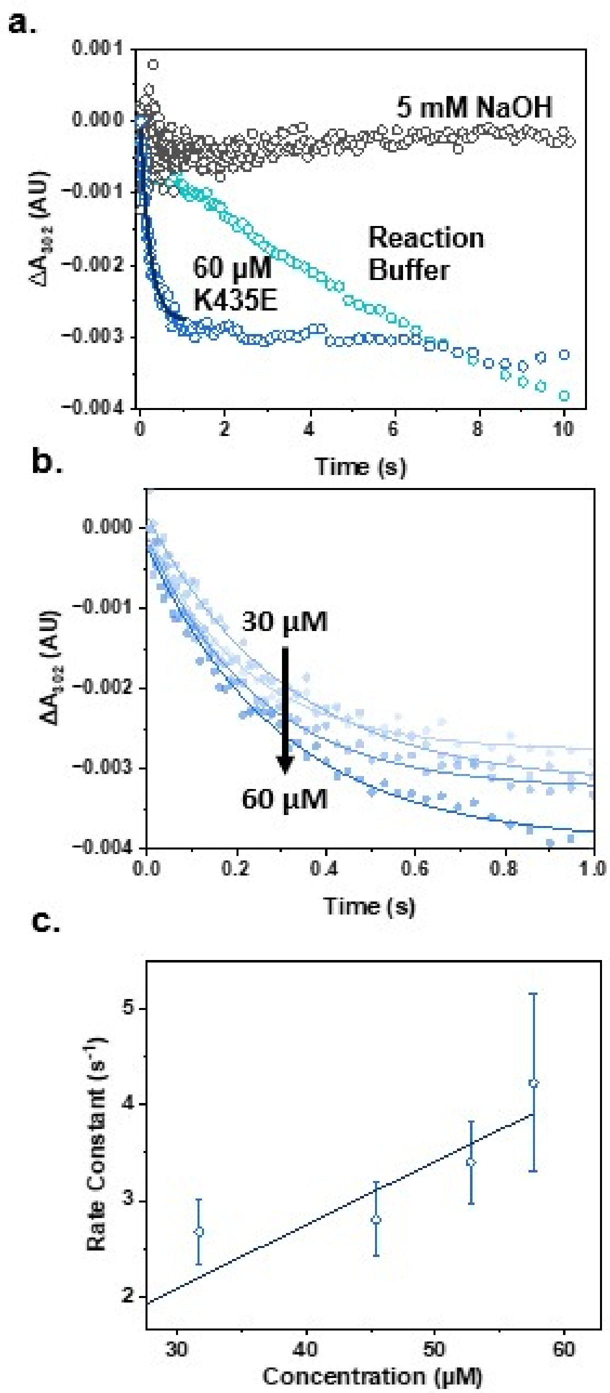

Figure S10. Peroxynitrite Decay of Mutant K435E. (a) Stopped flow spectra to follow the change in peroxynitrite concentration at 302 nm over time in the 5 mM NaOH (gray), reaction buffer (teal), and with 60 µM K435E in the reaction buffer (blue). Non-linear fitting is applied to the first second of reactivity (black). (b) Change in absorbance at 302 nm in the first second of K435E at various concentrations, with darker colors in the gradient blue corresponding to higher concentrations of protein, and their respective fits. (c) Pseudo-first-order rate constants for peroxynitrite reduction as a function of K435E concentration.

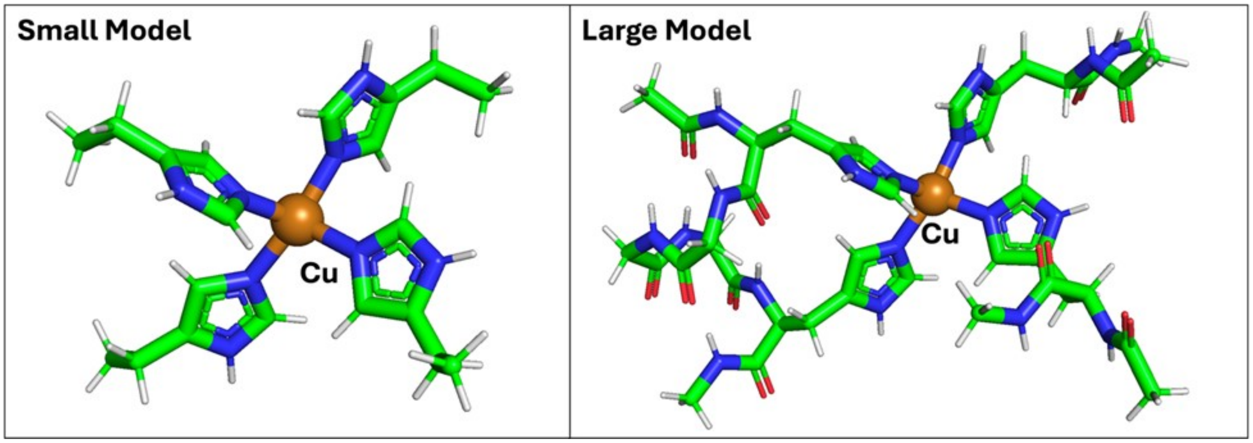

Figure S11. Optimized small and large models considered for DFT calculations. The small model was used to determine bond and angle parameters associated with the metal, with force constants derived using the Seminario method from the Hessian matrix obtained in DFT calculations. The large model was used for parameterizing partial charges through Merz-Kollman RESP calculations.

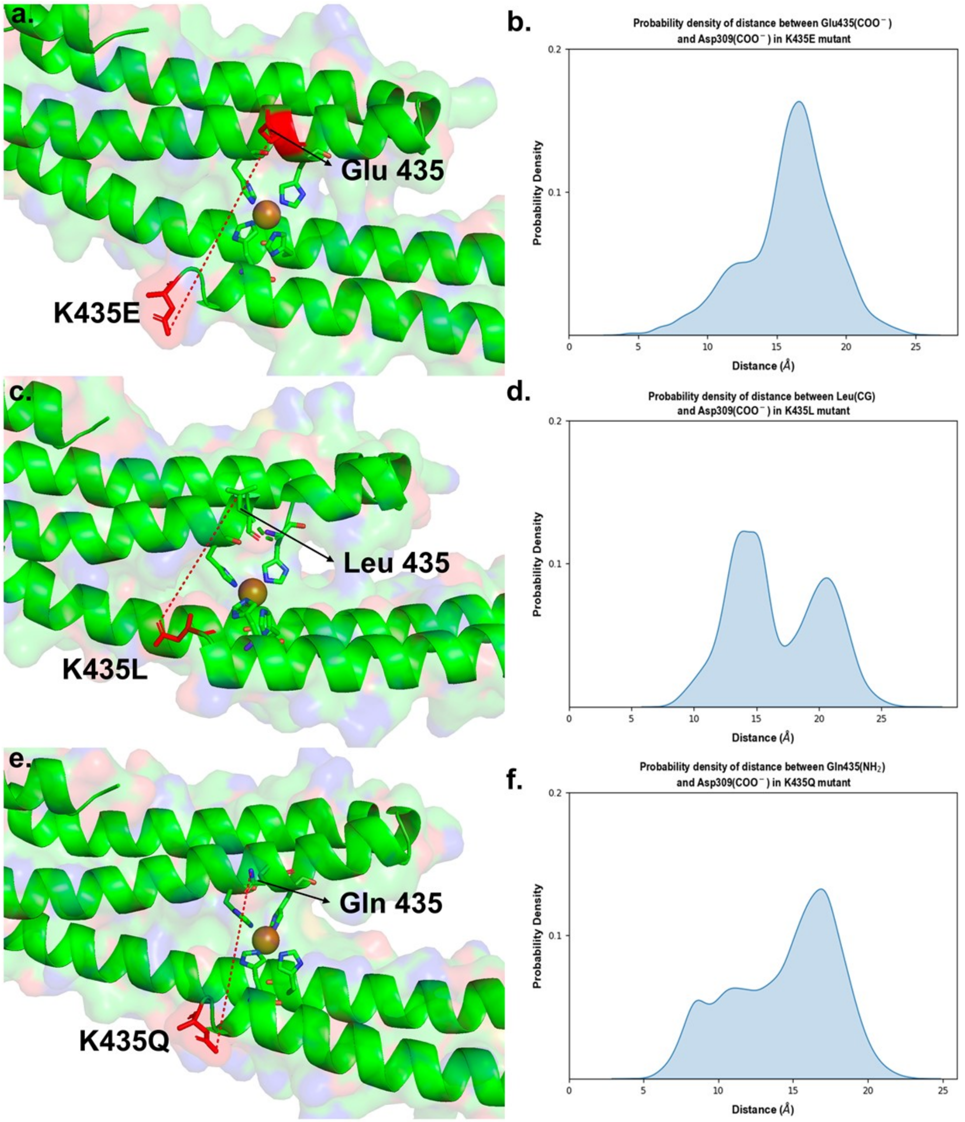

Figure S12. Molecular dynamics simulations of K435E, K435L and K435Q. The side chains of the K435 mutants do not interact with the carboxylate group of D309. These mutant side chains remain in the solvent, rotating freely, as indicated by the probability density analysis of the distance shown on the right.

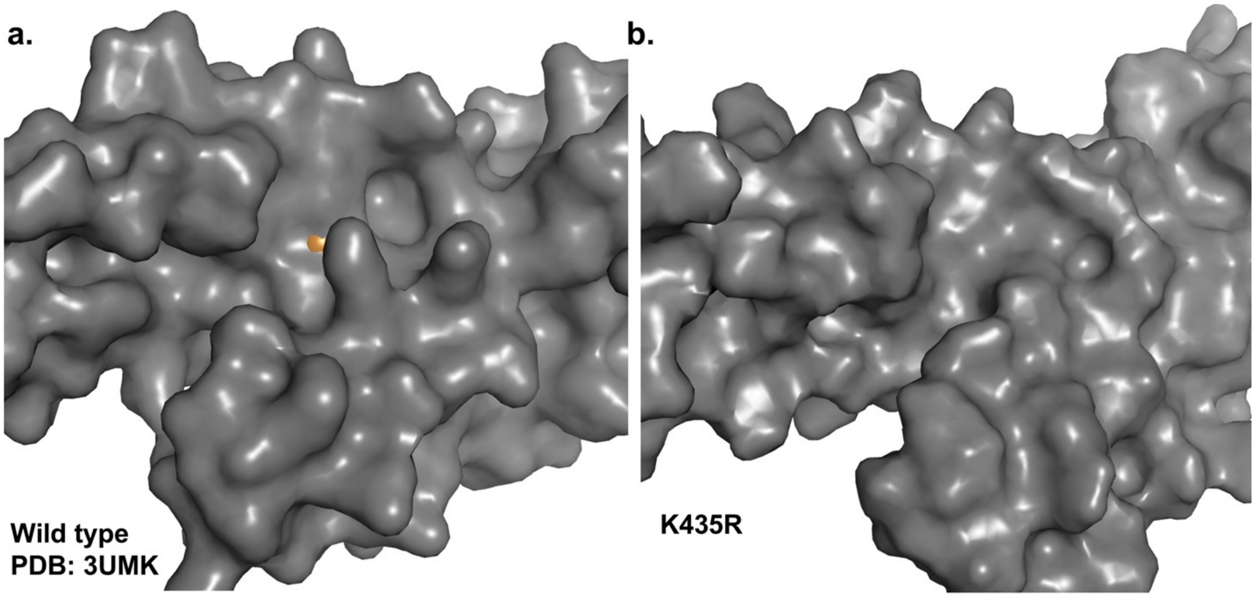

Figure S13. Surface map of wild-type Cu(II)-E2 compared to Cu(II)-K435R. (a) Surface representation of the crystal structure of wild-type Cu(II)-E2 (PDB: 3UMK) at van der Waals radius, with the surface shown in gray and the Cu(II) ion highlighted in orange. (b) Surface representation of the MD simulated structure of Cu(II)-K435R, generated using the same parameters. Newly formed hydrogen bonds obscure the Cu(II) ion (orange), making it inaccessible for visualization from all angles.
